# Island-sensitivity of two different interpretations of *why* in Chinese

**DOI:** 10.3389/fpsyg.2022.1059823

**Published:** 2023-01-20

**Authors:** Nayoun Kim, Ziying Li, Jiayi Lu

**Affiliations:** ^1^Department of English Language and Literature, Sungkyunkwan University, Seoul, South Korea; ^2^Department of Linguistics, Stanford University, Stanford, CA, United States

**Keywords:** experimental syntax, island effects, Chinese, *wh*-*in-situ*, argument-adjunct asymmetry

## Abstract

It has been assumed that the *wh*-element *weishenme* “why” in Chinese has two distinct interpretations: a reason reading, which typically yields *yinwei* “because”-answers, and a purpose reading, which typically triggers *weile* “in order to”-answers. It is claimed that the two interpretations differ in island sensitivity: the reason *weishenme* is sensitive to islands while the purpose *weishenme* is not. Assuming that the reason *weishenme* is a *wh*-adverb without finer internal structure, while the purpose *weishenme* is a *wh*-PP consisting of the preposition *wei* “for” and a *wh*-DP *shenme* “what,” this contrast in island sensitivity can be considered as an instance of a broader generalization: the so-called argument-adjunct asymmetry (or the DP-adverb asymmetry) of *wh*-*in-situ* island sensitivity. However, recent experimental studies provided mixed findings on whether the argument-adjunct asymmetry of *wh*-*in-situ* island sensitivity actually holds. The current study focuses on the two interpretations of *weishenme* “why/for what” in Chinese, and provides evidence using a formal acceptability judgment experiment that the two *weishenme*s are both sensitive to islands, contrary to previous generalizations. Our results provide further empirical challenge to the so-called argument-adjunct asymmetry of *wh*-*in-situ* island sensitivity.

## 1. Introduction

The *wh*-element *why* in Chinese, *weishenme*, has two distinct interpretations (Lin, 1992; Tsai, 1994, 1999, 2008; Stepanov and Tsai, 2008; Fujii et al., 2014): “reason *weishenme*” (henceforth *weishenme_R_*), which typically yields *yinwei* “because”-answers, as in (1a); and “purpose *weishenme*” (henceforth *weishenme_P_*), which typically triggers *weile* “in order to”-answers, as in (1b).

1. A: Xiaoli weishenme jingchang bangzhu Xiaochen?
Xiaoli why/for.what often help Xiaochen
a. “Why does Xiaoli often help Xiaochen?”
B: Yinwei ta xihuan Xiaochen.
because he like Xiaochen
“Because he likes Xiaochen.”
b. “For what purpose does Xiaoli often help Xiaochen?”
B. Weile taohao Xiaochen.
in.order.to please Xiaochen
“In order to please Xiaochen.”

It has long been assumed that the two interpretations of *weishenme* “why” in Chinese have different internal structures which may contribute to their island-(in)sensitivity (*cf*. Tsai, 1994): *weishenme_R_* is regarded as simply a *wh*-adverb without any internal syntactic structure, as in (2a); whereas *wei (le) shenme_P_* is considered a *wh-*PP consisting of the preposition *wei* “for” and its nominal part *shenme* “what,” where the suffix -le can be inserted between (e.g., Tsai, 1994; Stepanov and Tsai, 2008), as in (2b).

2. a. [_AdvP_ [*weishenme* “why”]].
b. [_PP_ [_P_ *wei(le)* “for”] [_DP_ *shenme* “what”]].

In addition to the difference in their internal structures, the two interpretations of *weishenme* are also claimed to differ in island sensitivity (Lin, 1992; Tsai, 1994; Stepanov and Tsai, 2008; Fujii et al., 2014). Consider examples (3–4).

3. [Women {a. *weishenme, b. wei(le)shenme} nianshu]
we why for what study
cai you yiyi?
just have meaning
a. *“What is the reason x such that it is meaningful [for us to study for x]?”
b. “What is the purpose x such that it is meaningful [for us to study for x]?”

4. Ni bijiao xihuan [[{a. *weishenme, b. wei(le)shenme}
you more like why for what
gongzuo] de ren]?
work REL people.
a. *“What is the reason x such that you like better [people [who work for x]]?”
b. “What is the purpose x such that you like better [people [who work for x]]?”

Tsai (1994): 128 (10–11)

As shown in examples (3) and (4), *weishenme_P_* is licensed inside island structures (i.e., insensitive to islands), whereas *weishenme_R_* is not (i.e., sensitive to islands). There are two main classes of accounts for this asymmetry.^1^ Some attribute it to the Empty Category Principle (ECP) while assuming that covert (LF) movements are not restricted by subjacency (Chomsky, 1981; Stowell, 1981; Huang, 1982). Others claim that *in-situ wh*-DPs, like the *shenme* “what” in (2b), do not need to undergo covert movement, and can be licensed *in-situ via* Unselective Binding (UB; Baker, 1970; Pesetsky, 1987; Nishigauchi, 1990; Aoun and Li, 1993; Tsai, 1994, 1999; Reinhart, 1998; Stepanov and Tsai, 2008). Both classes of accounts predict that *wh*-DPs are not sensitive to islands, while *wh*-adverbs are. Given the structural assumption in (2), *wei(le)shenme_P_* should be island insensitive because it is a PP containing a *wh*-DP, while *weishenme_R_* should be island sensitive because it is a *wh*-adverb.

This asymmetry in island sensitivity between *in-situ wh*-DPs and *wh*-adverbs mentioned above has been established mostly based on informal judgments by syntacticians. However, recent experimental studies on *wh*-*in-situ* languages put this generalization into question: Kim and Goodall (2016) found that Korean *wh*-DPs are sensitive to *wh*-islands; Omaki et al. (2020) found that Japanese *in-situ wh*-adverbs are equally insensitive to subject islands as *in-situ wh*-DPs; Lu et al. (2020) found that Chinese *wh*-DPs and *wh*-adverbs are both sensitive to relative clause islands.^2^ Crucially, Lu et al. (2020) pointed out that the acceptability contrasts between *wh*-DPs and *wh*-adverbs inside island structures (e.g., the contrast between (3/4a) and (3/4b)) was due to a penalty of long distance covert movement of *wh*-adverbs, which has nothing to do with island sensitivity. Similar bans on embedded *in-situ wh*-adverbs have also been proposed by Heycock (2006) and Jin (2016). If the island sensitivity asymmetry between *wh*-DP and *wh*-adverbs are indeed non-existent in Chinese, we should expect the two interpretations of *weishenme* to be equally sensitive (or insensitive) to island constraints. The current study thus follows Lu et al.’s (2020) general experimental design, and tests experimentally whether the two interpretations of *weishenme* indeed differ in island sensitivity.

## 2. Probing island effects experimentally

Island violation arises when there are two factors present in a sentence: an island construction (e.g., a relative clause, a clausal adjunct, etc.), and a syntactic dependency that crosses the boundary of the island construction.^3^ Given that island constructions and long distance dependencies might independently contribute to acceptability degradation, island violation should be detected as the superadditive effect of having both factors present at the same time (Sprouse et al., 2012; Sprouse and Hornstein, 2013; Sprouse and Villata, 2021). Following this argument, Sprouse et al. (2012) among others suggested that island effect can be probed experimentally using formal acceptability judgment tasks with a 2 × 2 factorial design, manipulating the structure of the embedded clause (island or non-island) and dependency distance (long or short, where long represents that the movement crosses the embedded clause boundary, and short represents that the movement does not cross the embedded clause boundary). An example set of stimuli is shown below in (5). Island effect would be detected as an interaction of embedded structure and dependency distance (i.e., the contrast between (5a) and (5b) is larger than the contrast between (5c) and (5d)).

5. Example stimuli for probing relative clause island effects with a 2 × 2 factorial design.
a. Embedded structure is island, long extraction:
What did John see the girl who was eating__?
b. Embedded structure is island, short extraction:
Who __ saw the girl who was eating sushi?
c. Embedded structure is non-island, long extraction:
What did John think that the girl was eating __?
d. Embedded structure is non-island, short extraction:
Who __ thought that the girl was eating sushi?

This design can also be used to probe the island sensitivity of *in-situ wh*-elements where there is no overt movement (Sprouse et al., 2011; Kim and Goodall, 2016; Lu et al., 2020). In such cases, the dependency distance factor represents whether the dependency between the *wh*-*in-situ* and its scope position crosses the embedded clause boundary or not. Similar stimuli sentences as (5) could be used in such a study, except that the gap positions in (5) would be occupied by *in-situ wh*-elements.

Note that in this paradigm, the existence of an island effect does not depend on the absolute acceptability rating of the island/long extraction condition [example (5a)]. It is possible that sentences like (5a) are rated as acceptable and receive no asterisk in introspective judgments, yet an acceptability judgment experiment might still detect a significant interaction of embedded structure and extraction distance, suggesting the existence of an island effect.^4^

In this study, we will use the same factorial design to probe the island sensitivity of *weishenme_R_* (the Reason *why*) and *weishenme_P_* (the Purpose *why*) in Chinese.

### 2.1. Predictions based on previous generalizations

Following the previous generalization by Tsai (1994) among others, *weishenme_R_* needs to undergo covert LF movement and is predicted to show island sensitivity. By contrast, *weishenme_P_*, which, just like *weileshenme_P_*, is a *wh*-PP and contains a nominal *wh*-element that can be licensed *in-situ* without covert movement, should be insensitive to islands. Using the factorial design to probe for island sensitivity as introduced in the previous sections, we expect there to be a significant interaction of embedded structure and dependency distance when the *wh*-element is *weishenme_R_* but not *weishenme_P_* or *weileshenme_P_*.

### 2.2. Method

To test our hypothesis, we conducted an acceptability judgment experiment employing a 2 × 2 × 3 design, manipulating the **structure** of the embedded clause (non-RC vs. RC, where “non-RC” refers to a complement clause structure, and “RC” refers to a relative clause structure), *wh*-scope dependency **distance** (short vs. long, where “short” means the *wh*-scope dependency does not cross the embedded clause boundary, and “long” means the *wh*-scope dependency crosses the embedded clause boundary), and ***wh*-type** (*weishenme_R_*, *weishenme_P_*, and *weileshenme_P_ ‘for what’*). Note that we followed Lu et al. (2020) and used relative clauses to probe for island sensitivity. All stimuli are in the form of question-answer pairs. The question sentence for the *weishenme_R_* and the *weishenme_P_* conditions are identical, and the difference in *wh*-type is achieved through the answer sentences that disambiguate the intended interpretation of *weishenme* in the question sentence. The *weileshenme_P_* “*for what*” condition is included as a sanity check: its question sentences are the same as the other two conditions except that the *wh*-element *weishenme* “why” is replaced with *weileshenme* “for what,” forcing the purpose interpretation. Example stimuli are shown below in Table 1.

**Table 1:** Example stimuli for the experiment.

	Non-RC		
	*weishenme_R_*	*weishenme_P_*	*weileshenme_P_*
Short	A: Anna weishenme shuo Liming Anna why say Liming taoke le? skip.class PERF “Why did Anna *t* say Liming skipped the class?” B: Yinwei Liming jintian mei because Liming today not lai shangke. come have.class“Because Liming did not come to class today.”	A: Anna weishenme shuo Liming Anna for.what say Liming taoke le? skip.class PERF “For what purpose did Anna *t* say Liming skipped the Class?” B: Weile pohuai Liming in.order.to destroy Liming de mingsheng. REL reputation “In order to destroy Liming’s reputation.”	A: Anna weileshenme shuo Liming Anna for.what say Liming taoke le? skip.class PERF “For what purpose did Anna *t* say Liming skipped the Class?” B: Weile pohuai Liming in.order.to destroy Liming de mingsheng. REL reputation “In order to destroy Liming’s reputation.”
Long	A: Anna shuo Liming weishenme Anna say Liming why taoke le? skip.class PERF “Why did Anna say Liming *t* skipped the class?” B: Ta shuo Liming yinwei bu she say Liming because not xihuan na men ke suoyi like that CL course so taoke le. skip.class PERF “She said that Liming skipped the class because he did not like that course.”	A: Anna shuo Liming weishenme Anna say Liming for.what taoke le? skip.class PERF “For what purpose did Anna say Liming *t* skipped the class?” B: Ta shuo Liming weile she say Liming in.order.to wan youxi taoke le. play game skip.class PERF “She said that Liming skipped the class in order to play games.”	A: Anna shuo Liming weileshenme Anna say Liming for.what taoke le? skip.class PERF “For what purpose did Anna say Liming *t* skipped the class?” B: Ta shuo Liming weile she say Liming in.order.to wan youxi taoke le. play game skip.class PERF “She said that Liming skipped the class in order to play games.”
	**RC**		
	***weishenme***_***R***_	***weishenme***_***P***_	***weileshenme***_***P***_
short	A: Anna weishenme ma le Anna why scold PERF taoke de xuesheng? skip.class REL student “Why did Anna *t* scold the student who skipped the class?” B: Yinwei ta tai shengqi le. because she too angry PERF “Because she was too angry.”	A: Anna weishenme ma le Anna for.what scold PERF taoke de xuesheng? skip.class REL student “For what purpose did Anna *t* scold the student who skipped the class?” B: Weile jiaoyu ta. in.order.to educate him “In order to educate him.”	A: Anna weileshenme ma le Anna for.what scold PERF taoke de xuesheng? skip.class REL student “For what purpose did Anna *t* scold the student who skipped the class?” B: Weile jiaoyu ta. in.order.to educate him “In order to educate him.”
long	A: Anna ma le weishenme Anna scold PERF why taoke de xuesheng? skip.class REL student “Why did Anna scold the student who *t* skipped the class?” B: Ta ma le na ge yinwei she scold PERF that CL because bu xihuan na men ke er not like that CL course so taoke de xuesheng. skip.class REL student “She scolded the student who skipped the class because he did not like that course.”	A: Anna ma le weishenme Anna scold PERF for.what taoke de xuesheng? skip.class REL student “For what purpose did Anna scold the student who *t* skipped the class?” B: Ta ma le na ge she scold PERF that CL weile wan youxi er in.order.to play game so taoke de xuesheng. skip.class REL student “She scolded the student who skipped the class in order to play games.”	A: Anna ma le weileshenme Anna scold PERF for.what taoke de xuesheng? skip.class REL student “For what purpose did Anna scold the student who *t* skipped the class?” B: Ta ma le na ge she scold PERF that CL weile wan youxi er in.order.to play game so taoke de xuesheng. skip.class REL student “She scolded the student who skipped the class in order to play games.”

The experiment was implemented on PC IbexFarm, a web-based presentation platform (Drummond, 2020). A total of 40 native speakers of Chinese (age range: 20–40, mean age: 24) were recruited to participate in the experiment. Each participant was paid an electronic convenience store voucher with the equivalent value of ₩2000 (approximately $2) after completion of the experiment. All participants were born in mainland China, acquired Mandarin Chinese as their first language, and use Chinese as a dominant language. They participated in the experiment using their own laptop *via* the experimental link distributed through *Prolific*.*co*. Experimental stimuli (Question/Answer pairs) were presented one at a time, and participants were asked to rate the naturalness of the Question/Answer pair (1 = totally unnatural, 7 = totally natural). A total of five practice questions were given prior to the actual experiment. There were 16 critical items in total. Each critical item appears exactly once for each participant, and randomly appears as one of the critical conditions. Also included in each presentation list were 48 filler items irrelevant to the current experimental manipulation. Among the filler items, there are 24 natural question-answer pairs and 24 unnatural ones. The fillers contain yes-no questions and *wh*-questions (other than *weishenme* “why”) with no island structures. Each participant saw 64 test items in total in addition to the 5 practice items at the beginning of the experiment.

### 2.3. Results

**Figure 1:**
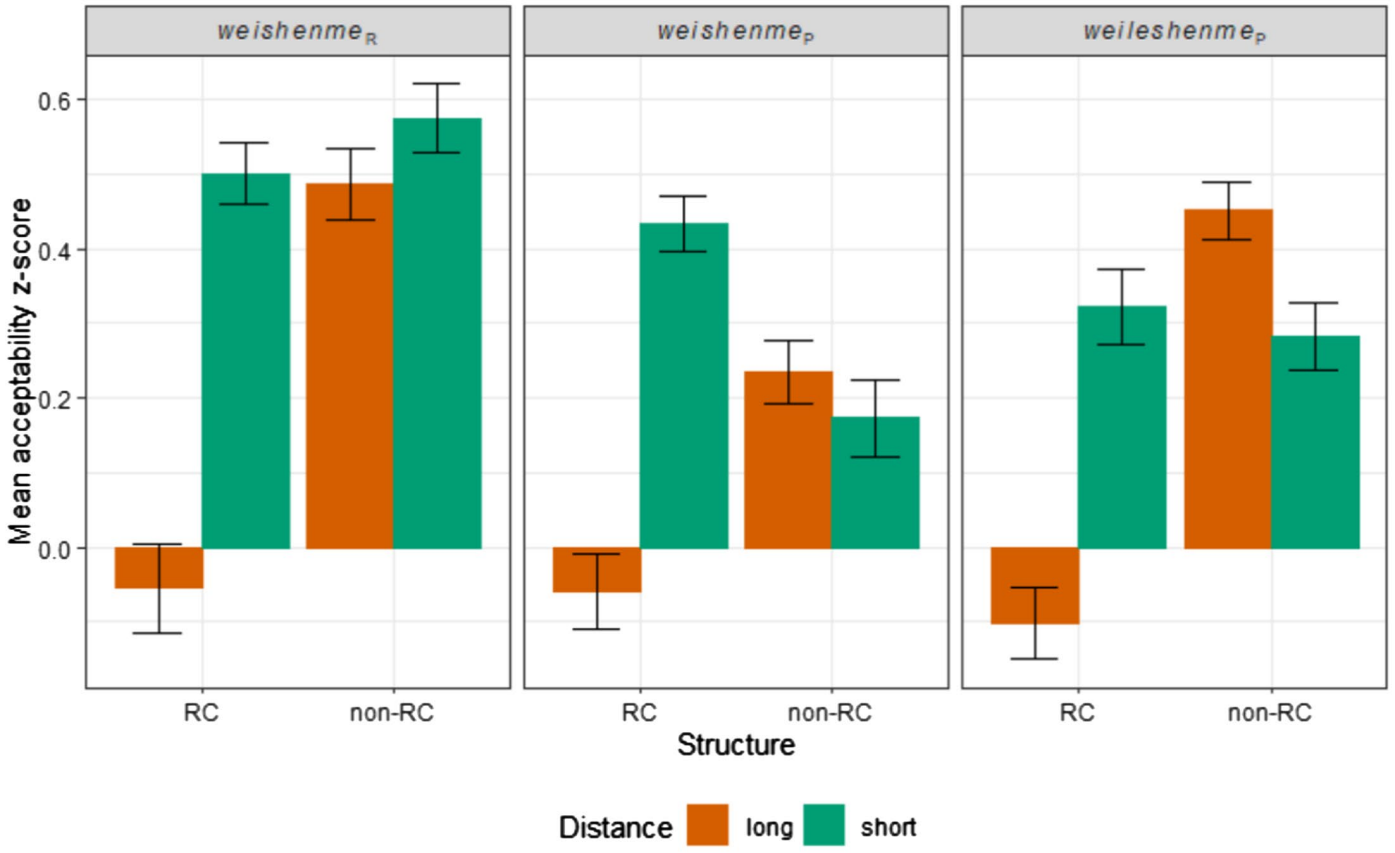
Mean acceptability Z-scores for all conditions.

We first calculated the by-participant z-score from the raw ratings. Among the filler items, natural fillers received a mean acceptability z-score of 0.67 (SE = 0.021), and unnatural fillers received a mean acceptability z-score of-0.91 (SE = 0.022). The mean acceptability z-score of each critical condition is shown in Figure 1. For each of the three *wh*-types (*weishenme_R_*, *weishenme_P_*, and *weileshenme_P_*), we analyzed the results using the same linear mixed-effects regression model (Baayen et al., 2008; Barr et al., 2013), predicting acceptability rating from the sum-coded fixed effects of Structure (non-RC vs. RC) and Distance (short vs. long) and their interaction. Also included in each model are the by-participant and by-item random intercepts and random slopes for both fixed effects and their interaction. Island effects, as discussed earlier, is detected as a positive interaction between Structure and Distance.

In the *weishenme_R_* condition, we found significant main effects of Structure (β = 0.12, SE = 0.057, *t* = 2.20) and Distance (β = 0.16, SE = 0.078, *t* = 2.10) such that the non-RC condition is more acceptable than the RC condition, and the short condition is more acceptable than the long condition. Furthermore, there is a significant interaction between Structure and Distance (β = 0.11, SE = 0.051, *t* = 2.24).

In the *weishenme_P_* condition, there is no significant main effect of Structure (β = 0.012, SE = 0.048, *t* = 0.26), but there is a marginally significant main effect of Distance (β = 0.11, SE = 0.062, *t* = 1.77) such that the short condition is more acceptable than the long condition. There is also a significant interaction between Structure and Distance (β = 0.16, SE = 0.060, *t* = 2.65).

In the *weileshenme_P_* condition, there are significant main effects of Structure (β = 0.15, SE = 0.057, *t* = 2.55) and Distance (β = 0.090, SE = 0.044, *t* = 2.07) such that the non-RC condition is more acceptable than the RC condition, and the short condition is more acceptable than the long condition. There is also a significant interaction between Structure and Distance (β = 0.15, SE = 0.047, *t* = 3.29).

To test whether there is any difference between the *wh*-types in terms of island sensitivity, we pooled the data from all three *wh*-types together and fit a linear mixed-effects regression model predicting acceptability rating from the sum-coded fixed effects of Structure and Distance, and the dummy-coded fixed effect of *Wh*-type with reference level set to *weishenme_P_*. The model also includes by-item and by-participant random intercepts and random slopes for all three fixed effects and their interactions. We observed no significant three-way interaction for either *weishenme_R_* (β = 0.027, SE = 0.066, *t* = 0.41) or *weileshenme_P_* (β = −0.016, SE = 0.066, *t* = −0.25). An omnibus test (Type III ANOVA with Satterthwaite’s method) on the model shows that there is no significant three-way interaction (*F*(2) = 0.083, *p* = 0.92), further confirming that *wh*-type does not affect the magnitude of islandhood.

## 3. Discussion

Contrary to our hypothesis, we found significant interactions of Structure and Distance for all three *wh*-types tested, suggesting that all three types of *wh*-elements (*weishenme_R_*, *weishenme_P_*, and *weileshenme_P_*) are sensitive to the relative clause island. This challenges previous empirical generalizations that *weishenme_P_* and *weileshenme_P_* are not island sensitive. Note that despite the significant interactions of Structure and Distance, the long extraction/RC structure conditions may not be perceived as unacceptable due to their middle-of-the-scale absolute ratings. The long/RC/*weishenme_R_* condition received an acceptability z-score of-0.054 (SE = 0.12), the long/RC/*weishenme_P_* condition received an acceptability z-score of-0.059 (SE = 0.099), and the long/RC/*weileshenme_P_* condition received an acceptability z-score of-0.10 (SE = 0.095). They were all rated higher than the unnatural fillers, which received an acceptability z-score of-0.91 (SE = 0.022). The high ratings for the long/RC conditions may have contributed to the misguided empirical generalizations in the past literature that were built upon introspective judgments.

Furthermore, no three-way interaction of Structure, Distance, and *Wh*-type is found when comparing the *weishenme_P_* condition with the other two *wh*-types. There are two conclusions we can draw from this finding. First, *weishenme_P_* and *weileshenme_P_* behave similarly with regard to island sensitivity, suggesting that our sanity check using *weileshenme_P_* yielded expected results. Second, *weishenme_P_* and *weishenme_R_* are equally restricted by the relative clause island, contrary to previous generalizations. Admittedly, we are arguing from the lack of an effect which could be due to a lack of power. However, we should note that the three-way interaction effect is numerically in the direction that the interaction effect between Structure and Distance is larger in the *weishenme_P_* condition than in the *weishenme_R_* condition, opposite of what previous generalizations predict even if it is a false negative.

In sum, the results of the current study show that both the reason and the purpose interpretations of *weishenme* “why,” as well as the *wh*-PP *weileshenme* “for what” are all restricted by the relative clause island in Chinese. This poses a challenge to various syntactic accounts of Chinese *wh*-*in-situ*. Assuming that the *weishenme_P_* and *weileshenme_P_* both have the internal structure in (2b), repeated below as (6), they both contain a *wh*-DP *shenme* “what” (Lin, 1992; Tsai, 1994, 1999, 2008; Stepanov and Tsai, 2008; Fujii et al., 2014). Therefore, they should be grammatical when appearing inside an island according to theories that assume that island effects (or to be more specific, subjacency requirements) do not operate at the LF level (Huang, 1982; Pesetsky, 1987; Uriagereka, 1999; Fox and Pesetsky, 2005), and theories that assume *in-situ wh*-DPs do not need to undergo LF movement to its scope position (Baker, 1970; Pesetsky, 1987; Nishigauchi, 1990; Tsai, 1994; Reinhart, 1998).

6. [_PP_ [_P_ *wei(le)* “for”] [_DP_ *shenme* “what”]].

However, we saw in the experiment that island violations arise when *in-situ weishenme_P_* and *weileshenme_P_* appear inside relative clauses. This suggests that either the structure in (6) is incorrect and both *weishenme_P_* and *weileshenme_P_* are in fact *wh*-adverbs just like *weishenme_R_*, or that *wh*-DPs also undergoes LF movement while island effects restrict such movements. The latter approach is supported by recent experimental findings (Lu et al., 2020) that the *in-situ wh*-DP *shenme* “what” is in fact restricted by islands contrary to previous claims, and fits nicely with developments in minimalist syntax that covert and overt movements are essentially the same with the only difference being which copy of the moved element is pronounced (Chomsky and Howard, 1993; Nunes, 1995, *inter alia*), and should thus be subject to the same set of restrictions.

One interesting observation pointed out by an anonymous reviewer is that the *weishenme_P_* conditions are generally rated to be less acceptable than the *weishenme_R_* conditions. Although we do not have a definitive explanation for this contrast, below are two possibilities. First, in the current experimental design, *weileshenme_P_* is a salient lexical alternative for *weishenme_P_*, while *weishenme_R_* lacks such an alternative. Through Gricean reasoning, when the word *weishenme* is used, the comprehenders would infer that the intended meaning is more likely a reason interpretation, because the unambiguous *weileshenme_P_* could have been used had the intended meaning been a purpose interpretation. Another possibility is that the purpose interpretation of *weishenme* is simply less frequent compared to the reason interpretation. This possibility could be tested by further corpus studies.

## 4. Conclusion

In this study, we provide experimental evidence that both the reason and purpose interpretations of *weishenme* “why,” and the *wh*-PP *weileshenme* “for what” are all sensitive to island effect in Chinese. Furthermore, we found no evidence suggesting any difference in their island sensitivity. These results challenge the longstanding generalization that nominal *wh*-in-situ are island insensitive in Chinese.

## Data availability statement

The raw data supporting the conclusions of this article will be made available by the authors, without undue reservation.

## Ethics statement

The studies involving human participants were reviewed and approved by Sungkyunkwan University. The patients/participants provided their written informed consent to participate in this study.

## Author contributions

NK and JL conceived the study, implemented the experiment, conducted the statistical analyses of the data, and supervised the stimuli creation. ZL created the stimuli. All authors contributed to planning the research and participated in writing the article.

## Funding

This research was supported by the Sungkyunkwan University and the BK21 FOUR (Graduate School Innovation) funded by the Ministry of Education (MOE, South Korea) and National Research Foundation of Korea (NRF).

## Conflict of interest

The authors declare that the research was conducted in the absence of any commercial or financial relationships that could be construed as a potential conflict of interest.

## Publisher’s note

All claims expressed in this article are solely those of the authors and do not necessarily represent those of their affiliated organizations, or those of the publisher, the editors and the reviewers. Any product that may be evaluated in this article, or claim that may be made by its manufacturer, is not guaranteed or endorsed by the publisher.

## Footnotes

^1^See Cheng (2009) for a comprehensive review on the various analyses of *in-situ wh*-elements.

^2^See Tian et al. (2022) for an argument that Lu et al. (2020) experimental design is confounded.

^3^The terms "island," "island violations," and "islandhood" are used in this paper in a purely descriptive and theory-neutral way: we remain agnostic about whether such "island violations" are the result of violated grammatical constraints, or the result of processing-level or informational structural constraints. In the current study, we simply probe for the island sensitivity of *wei(le)shenme* in Chinese with the aim to verify/challenge previous empirical generalizations. For various non-grammar-level proposals for islands effects in general, see Chaves (2021) and Liu et al. (2022), *inter alia*.

^4^The high absolute ratings of island/long-extraction sentences might point to processing-level or discourse-level accounts of the island effect. However, for the purpose of discussion and in a purely descriptive sense, we still refer to such sentences as containing an "island violation" in this paper.
